# The efficacy of curcumin/Qing Dai combination in children with active ulcerative colitis: a multicenter retrospective cohort study

**DOI:** 10.3389/fped.2024.1342656

**Published:** 2024-09-27

**Authors:** Nurit Loberman Nachum, Nir Salomon, Anat Yerushalmy-Feler, Yael Weintraub, Dotan Yogev, Maya Granot, Yael Haberman, Shomron Ben-Horin, Batia Weiss

**Affiliations:** ^1^ Pediatric Gastroenterology and Nutrition Unit, Edmond and Lily Safra Children’s Hospital, Tel Hashomer, Israel; ^2^ Sackler Faculty of Medicine, Tel Aviv University, Tel-Aviv, Israel; ^3^ Department of Gastroenterology, Sheba Medical Center, Ramat Gan, Israel; ^4^ Pediatric Gastroenterology Institute, Dana-Dwek Children’s Hospital, Tel Aviv Sourasky Medical Center, Tel Aviv, Israel; ^5^ Institute of Gastroenterology, Nutrition and Liver Diseases, Schneider Children’s Medical Center of Israel, Petach Tikva, Israel; ^6^ Juliet Keidan Institute of Pediatric Gastroenterology and Nutrition, Shaare Zedek Medical Center, Jerusalem, Israel; ^7^ The Faculty of Medicine, The Hebrew University of Jerusalem, Jerusalem, Israel; ^8^ Department of Pediatrics, Cincinnati Children’s Hospital Medical Center, University of Cincinnati College of Medicine, Cincinnati, OH, United States

**Keywords:** Qing Dai, curcumin, ulcerative colitis, pediatric, management

## Abstract

**Background:**

Curcumin and Qing Dai (QD) are herbal extracts that recently showed efficacy in treating inflammatory bowel disease (IBD). Since 2016, a combination of curcumin with QD (CurQD) has been employed in our center for management of active ulcerative colitis (UC).

**Objectives:**

We report the effectiveness and safety of CurQD therapy in children with mild-moderate UC or IBD-unclassified (IBD-U).

**Design:**

A multicenter retrospective study.

**Methods:**

Children aged ≤OP18 years who were treated with CurQD during 2017–2021 were included. Disease activity measures were Pediatric UC Activity Index (PUCAI), and fecal calprotectin (FC). The primary outcome was a decrease in PUCAI by ≥10 points, FC normalization (≤100 µg/gr when baseline ≥300 µg/gr) or a ≥ 50% decrease in FC.

**Results:**

Of 30 patients (60% males, mean age 14 ± 3.9 years), 15 (50%), 13 (43%), and 2 (7%) had pancolitis, left-sided colitis and proctitis, respectively. The daily medication dose was 0.5–3 gm QD with 1–4 gm curcumin. Concomitant treatment at induction was corticosteroids (19%), biologics (28%) and 5-aminosalicylic acid (40%). The mean duration of induction was 11.6 weeks [95% confidence interval (CI) 10.2–13.1, range 8–16]. PUCAI decreased from a mean of 31.3 (95% CI 26.6–36.0, range 5–60) to 10.9 (95% CI 7.6–14.4, range 5–35) (*n* = 26, *p* < 0.001). FC response and normalization occurred in 11/12 and 7/12 patients, respectively. The median decline in FC was from 749 µg/gm [interquartile range (IQR) 566–1000] to 39 µg/gm (IQR 12–132) (*n* = 15, *p* = 0.04). During follow-up (median 8 months, IQR 6–10), 10 patients (33%) flared; five of them regained remission or responded to a treatment change. Of 18 patients treated beyond induction, 12 (67%) achieved clinical response and 10 achieved clinical remission by the end of follow up.

**Conclusion:**

CurQD may be effective and safe as an add-on option to conventional management, for induction and maintenance in children with mild-moderate UC/IBD-U.

## Introduction

Ulcerative colitis (UC) is a chronic inflammatory disease of the colonic mucosa, extending from isolated rectal disease to pancolitis. While the armamentarium for management of active UC has increased, novel compounds that may provide effective and safe treatment should still be explored, especially for the pediatric population (1–4).

Qing Dai (QD), also known as Indigo Naturalis, is a traditional Chinese medicine, derived from dried leaves and stems of blue flowers such as Baphicacanthus cusia Bremek, Polygonum tinctorium Ait and Isatis indigotica Fortune (5). QD has been used in traditional Chinese medicine for many years, due to its hemostatic, antipyretic, anti-inflammatory, sedative, antibacterial, antiviral, antioxidant and anti-tumor activities (6). QD has been shown to reduce inflammation through activation of the aryl hydrocarbon receptor (AhR), which promotes up-regulation of IL-22 and IL-10 (7). QD has been described as efficacious for treating inflammatory diseases like psoriasis (5, 8) and UC (9, 10).

A number of investigators have described the efficacy of a gut-directed formulation of curcumin in inducing and maintaining remission in patients with mild to moderate active UC (11, 12). Since 2016, a combination of curcumin with QD (CurQD) has been employed in our center, for management of active UC (13, 14). A recently published randomized placebo-controlled trial showed the efficacy of this combination in adults with active UC (15). However, data on curcumin or QD treatment in pediatric UC are scarce. Therefore, we aimed to report the efficacy and safety of CurQD management in a cohort of pediatric patients with UC or inflammatory bowel disease unclassified (IBD-U) who failed previous management.

## Materials and methods

### Patients

We conducted a multicenter retrospective cohort study of children treated at four pediatric gastroenterology units: Sheba Medical Center, Dana Dwek Children's Hospital, Schneider Children's Medical Center and Shaare Zedek Medical Center. Patient inclusion criteria were age ≤ 18 years; a Pediatric UC Activity Index (PUCAI) indicating mild or active UC or IBD-U (in the range of 10–35 and 40–60, respectively); and management with CurQD during 2017–2021. All the patients received CurQD at Sheba Medical Center, but clinical and endoscopic follow-ups were performed at each center. Clinical, laboratory and endoscopic data were retrieved from the medical records. Disease activity was assessed by the PUCAI (16) and by fecal calprotectin (FC) (17). We excluded from the analysis, patients who received less than 2 weeks of CurQD treatment, patients with missing significant data, and patients with loss of follow up.

### Management regimen

CurQD is produced by Evinature (Binyamina, Israel) in a Good Manufacturing Practice facility. This management was provided to all the patients by a trained therapist of Chinese medicine, at the Gastroenterology Department. The doses were in the range of 0.5–3 gm/day for QD and 1–4 gm/day for curcumin, depending on the severity of the disease activity and the patient's weight, and based on published literature. While gut-directed curcumin was demonstrated to be effective in mild-moderate active UC (11), Qing Dai was shown to be effective for moderate-severe UC (18). Therefore, varying the ratios between these two compounds enables personalizing CurQD administration to each patient's condition. For example, a patient presenting with mild-moderate disease activity received CurQD with a lower QD dose and a higher curcumin dose. In contrast, a patient with more active disease received a higher QD and lower curcumin dose. The dose range was adjusted to pediatrics by slightly lowering the dose described in the above-cited adult studies. The CurQD management regimen generally followed an induction dose of CurQD for 16 weeks, after which the dose of QD was gradually reduced, until only gut-directed curcumin was continued. For patients who flared during the tapering, the minimal dose of QD within the combination that was needed to control the disease was continued.

### Outcome

The primary outcome was a clinical response after CurQD induction, up to 16 weeks from the beginning of management. This response was defined as a decrease in PUCAI by at least 10 points, and reduction in FC levels by 50%. Endoscopic MAYO scores prior to and post treatment were compared when available. The secondary outcome was remission (PUCAI ≤ 10; FC ≤ 100, considering a baseline ≥300). Treatment failure was considered as discontinuation of CurQD due to adverse events or lack of response, hospitalization or induction of a biologic treatment or corticosteroids. When available, data regarding therapy beyond week 16 and until the end of the follow up were extracted. Concurrent therapy was given at the discretion of the treating physician.

### Statistical analysis

Nonparametric data are presented as means with standard deviations or confidence intervals, or as medians and interquartile ranges (IQR). A Mann-Whitney test was used to analyze differences between nonparametric data. Missing data were considered as non-available.

The study was approved by the ethics committees of all the institutions (No. 8998-21-SMC).

## Results

### Patients and management

The 30 patients included were with mild (PUCAI 10–35) or moderate (PUCAI 40–60) active UC, or IBD-U at recruitment. Eighteen (60%) were males. Eleven patients were from Sheba Medical Center, nine from Dana Dwek Children's Hospital, six from Schneider Children's Medical Center and four from Shaare-Zedek Medical Center. Table 1 presents the demographic and clinical characteristics of the study group. The mean age at CurQD initiation was 14 ± 3.9 years, and the median disease duration until CurQD treatment was 1.5 years (IQR 1.0–2.6). Disease extent was pancolitis in 15 (50%), left-sided colitis in 13 (43%) and proctitis in two (7%) patients. Eighteen patients (60%) were naïve to biologic treatment, and seven (22%) received more than one biologic therapy. At the time of CurQD induction, 19 (63%) patients were treated with 5-aminosalicylic acid, of them 13 as a single treatment. Six (20%) patients were treated with corticosteroids and six (20%) with immunomodulators. Nine (30%) patients were with active disease while receiving biologic treatment (4 Infliximab, 4 Vedolizumab and 1 Golimumab), to which the herbal compound was then added. The daily QD dose within the CurQD ranged from 0.5 to 3 gm, according to disease severity, combined with 1–4 gm of gut-directed curcumin.

**Table 1:** Demographic and clinical characteristics of pediatric patients with ulcerative colitis.

Age at diagnosis (years)	
Mean (SD)	12.17 (3.73)
Median (IQR)	13.15 (11–14)
Age at CurQD initiation (years)	
Mean (SD)	14 (3.89)
Median (IQR)	14.4 (12–16.5)
Gender	
Male (%)	18 (60)
Diagnosis (%)	
UC	28 (93)
IBD-U	2 (7)
Disease location (%)	
Proctitis	2 (7)
Left sided	13 (43)
Pancolitis	15 (50)
Previous treatments	
5ASA	29
Corticosteroids	19
Immunomodulators	8
Infliximab	10
Adalimumab	1
Vedolizumab	6
Golimumab	2
Concomitant treatment	
5ASA	19
Corticosteroids	6
Immunomodulators	6
Infliximab	4
Vedolizumab	4
Golimumab	1
Biologic treatment (%)	
None	20 (66)
1 Biologic	5 (17)
>1 Biologic	5 (17)
Treatment duration (months)	
Mean (SD)	12.9 (10.9)
Median (IQR)	11 (6–14)

UC, ulcerative colitis; IBD-U, inflammatory bowel disease unclassified; 5ASA, 5-aminosalicylic acid; SD, standard deviation; IQR, interquartile range.

### The efficacy of induction

**Figure 1:**
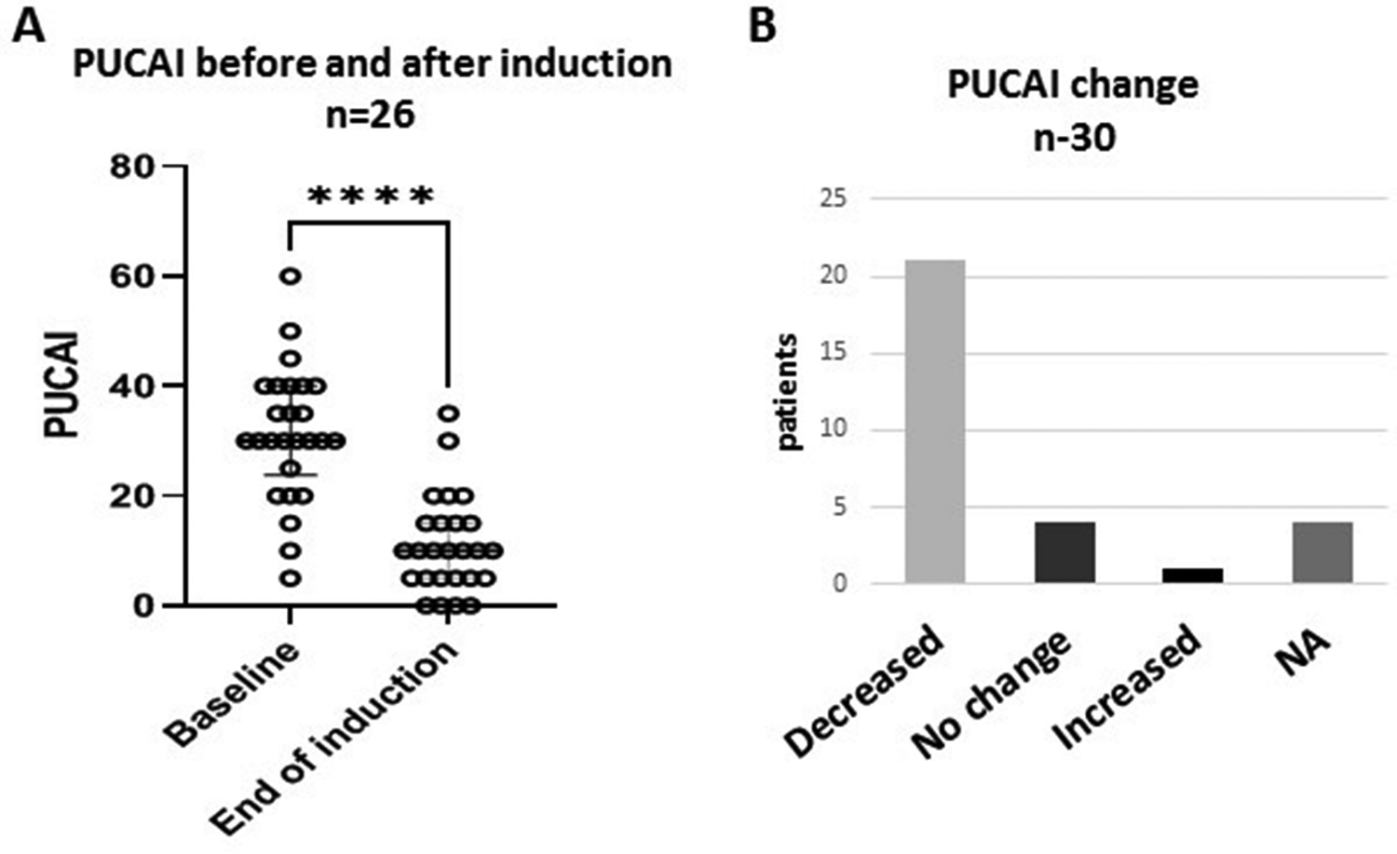
Clinical response to treatment. Changes in Pediatric Ulcerative Colitis Activity Index (PUCAI) scores after CurQD induction. **(A)** Decline in mean PUCAI from baseline. **(B)** Trends of PUCAI in the study group ****p < 0.0001; NA, not available.

PUCAI at baseline showed mild activity (≤35) in 18 (60%) and moderate activity (PUCAI 40–60) in 8 (27%) patients. The mean PUCAI score decreased from 31.3 (95% CI 26.6–36.0, range 5–60) at baseline, to 10.9 (95% CI 7.6–14.4, range 5–35), at the end of induction (Figure 1A). The mean duration of induction was 11.6 weeks (95% CI 10.2–13.1, range 8–16) (*p* < 0.001). After induction, PUCAI decreased in 21 patients, showed no change in four patients and increased in one patient (Figure 1B). *Of the 18 patients with mild disease, the PUCAI score decreased by >10 points in 13 (72%), was not changed in 4 (22%) and increased in 1 (6%). PUCAI decreased by >10 points in all eight patients with moderate disease and available data.* In four patients, PUCAI was available at baseline and not post induction.

**Figure 2:**
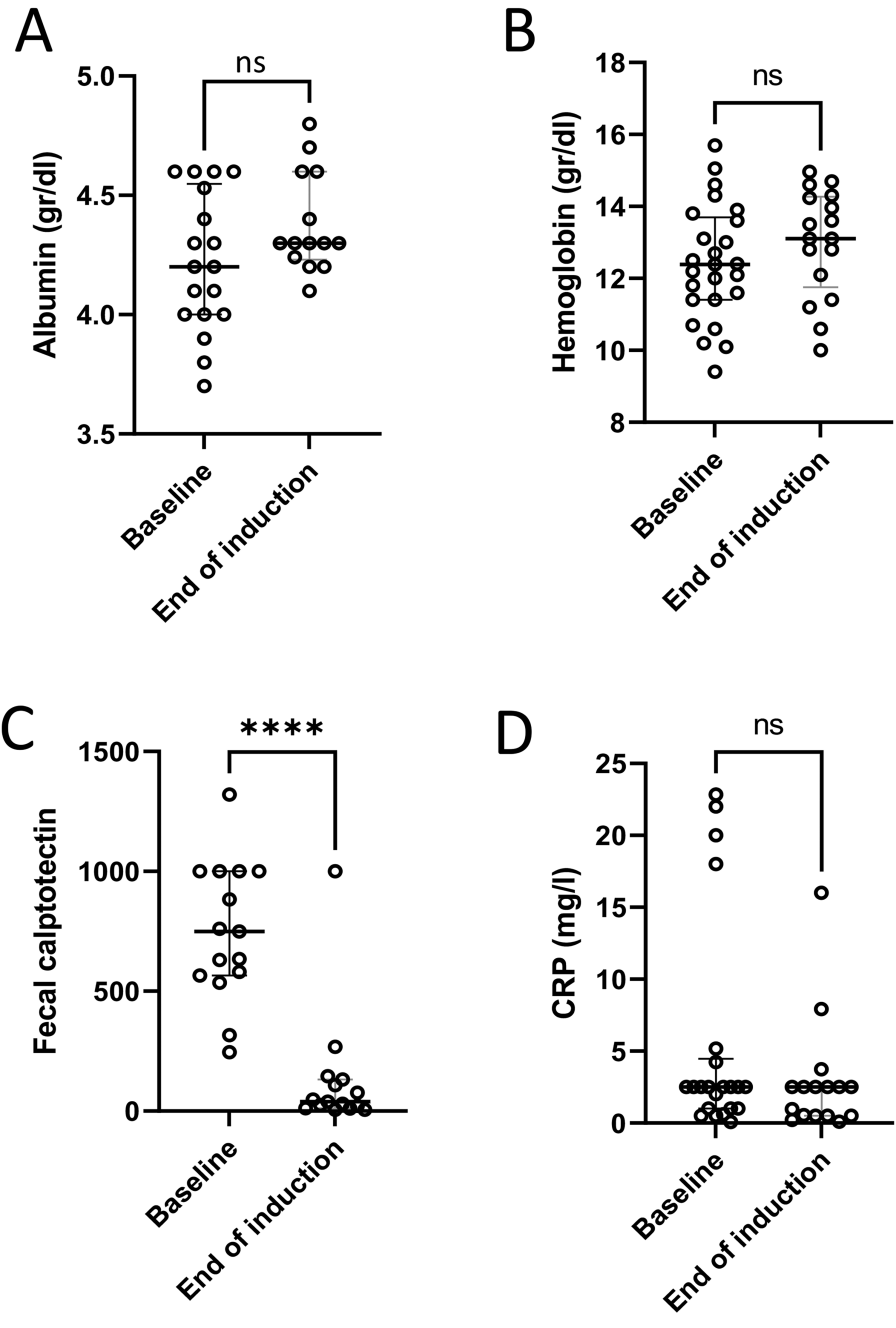
Laboratory response to treatment. Laboratory tests at baseline and at the end of induction showed no significant changes in serum albumin **(A)**, hemoglobin level **(B)** or C-reactive protein (CRP) **(D)**; and a significant decrease in fecal calprotectin **(C)** ****p < 0.0001, ns, non-significant.

Among the 12 patients with paired FC measurements before and after induction, the median value declined from 749 µg/gm (IQR 566–1000) to 39 µg/gm (IQR 12–132) (*p* = 0.04) (Figure 2C). Eleven (91%) of these patients had an FC response at the end of induction. For seven (64%) of them, FC at baseline was ≥300 µg/gm and reached normalization (≤100 µg/gm). Five patients had FC levels higher than 100 µg/gm. Two of them had a clinical flare with endoscopic evidence of inflammation, and one had only endoscopic evidence of inflammation without clinical signs. FC improved in six patients with mild disease and in three with moderate disease. No changes were observed in serum hemoglobin, albumin or C-reactive protein levels before and after induction (Figure 2).

Three (10%) patients discontinued CurQD treatment during induction due to lack of efficacy. One of them was treated for only 2 weeks, and two had clinical and endoscopic evidence of active disease and were treated with biologic therapy.

### Efficacy of maintenance

Twenty-seven patients continued to maintenance treatment; the mean follow-up time was 14.5 ± 11 months. Of the 21 who responded to induction, eight (33%) had a flare, after a mean of 12 (±10.6) months. One of these had a spontaneous remission with no additional treatment, one started 5-aminosalicylic acid medication, and one started budesonide with a good response. One patient temporarily discontinued CurQD, received antibiotics and renewed CurQD therapy after regaining remission. Another patient refused treatment escalation.

Data of paired FC at the end of the maintenance follow up were available for 13 patients. Twelve of them showed FC response, and five achieved normalization of FC. The median FC decreased from 749 (IQR 573–1,000) to 121.5 (IQR 35–174).

Colonoscopy was performed in eight (27%) patients during the follow-up period. Three of them had normal examinations and five had evidence of inflammation (Mayo score 2–3). One of the five patients improved clinically after the colonoscopy, while continuing management with CurQD only (Table 2).

**Table 2:** Endoscopic findings pre and post CurQD treatment and changes in treatment changes.

Patient	Previous treatment	Treatment	Lower endoscopy before QD treatment	Treatment change	Lower endoscopy after induction	Treatment change	Last lower endoscopy under QD treatment	Clinical outcome
1	5ASA, Steroids, Azathioprine, Infliximab, Adalimumab, Vedolizumab	Golimumab		CurQD			left-sided Mayo-3	No response - treatment change to Ustekinumab
2	5ASA, Steroids, Azathioprine, Infliximab	Infliximab Azathioprine	rectum- Mayo 2-3, descending and transverse Mayo 2	CurQD	rectum- Mayo 3, descending and transverse Mayo 1	change to Vedolizumab	normal	remission
3	5ASA, Steroids, MTX, Infliximab	5ASA	30 cm of ulcerated and hyperemic mucosa, Mayo 2	CurQD			30 cm of erythema	response
4	5ASA, Steroids, Azathioprine, Infliximab, Vedolizumab	Vedolizumab		CurQD, stop Vedolizumab			active colitis Mayo 2-3 until transverse colon	clinical improvement with CurQD only
5	5ASA, Steroids, Infliximab,	Infliximab	08/2019 sigmoidoscopy- colitis, Mayo 2-30 cm.	CurQD			normal	response
6	5ASA, Steroids, Infliximab, Vedolizumab	Vedolizumab5ASA		CurQD	severe pancolitis		severe pancolitis	No response
7	5ASA, Steroids, Azathioprine,	5ASA, Azathioprine		CurQD			active proctitis	No response
8	5ASA, Steroids, Infliximab,	None	Severe pancolitis	CurQD	severe left-sided colitis		active disease	No response

5ASA, 5-aminosalicylic acid, Cur, curcumin, MTX, methotrexate, QD, Qing Dai.

Based on the available data, during the follow up, 19/30 (63%) patients did not change their concomitant medications, five patients changed or added biologic treatment, and six patients stopped or added other medication.

### Safety

The adverse events included headache in one patient, which did not require any changes in medication and resolved spontaneously; and hematuria in one patient, which resolved after discontinuation of curcumin. During the maintenance treatment, one patient had elevated transaminases, up to three times the upper normal limit, while having a concomitant Covid-19 infection. Although it was not clear whether the increase in enzyme levels resulted from the viral disease or was a side effect of the treatment, CurQD was discontinued, and the enzymes improved.

No subgroup analyses were performed due to the small sizes of the groups.

## Discussion

Our findings showed potential efficacy and safety of CurQD management in children with UC or IBD-U who did not achieve complete remission with conventional management. Clinical response was achieved in 70% of the patients after induction; PUCAI and FC decreased significantly (Figures 1, 2C). Twelve of the 13 patients with available data showed a decrease in FC at the end of follow-up. We did not observe changes after induction in serum hemoglobin, albumin and C-reactive protein levels. This is probably because those markers were within normal limits in most of the patients prior to treatment induction.

In addition to efficacy, CurQD management seemed to be safe. The adverse events included only one patient with a spontaneously resolving headache, and one patient with elevated liver enzymes that co-existed with Covid-19 infection. That patient stopped treatment, and the liver enzymes normalized. However, it is not clear whether the increased levels were a side effect of the medication or due to the viral disease. We did not observe adverse effects that were previously reported for QD, such as gastrointestinal symptoms, liver dysfunction, cutaneous symptoms, leukopenia, dizziness and reversible pulmonary hypertension (18–21). We also did not observe even mild adverse events, as were reported in a clinical trial of curcumin, including abdominal distension, nausea and a transient increase in the number of bowel movements (12). One of our patients had hematuria, which is not among the side effects that have been reported for curcumin; the condition resolved after curcumin discontinuation.

The proposed mechanism of action of curcumin is reducing inflammation by downregulation of genes, such as PI3 K, related to oxidative stress and fibrogenesis pathways; and by decreasing the activity of proinflammatory cytokines like IFN-*γ*, TNF-α, IL-1 and IL-8 (22). QD reduces inflammation by activating AhR, which promotes up-regulation of IL-22 and IL-10 (7).

QD has been used in traditional Chinese medicine for centuries, for various infectious and inflammatory conditions (18). Several studies have examined the efficacy of QD management in adults and children with UC. In one of the first prospective studies, Sugimoto et al. (10) demonstrated significant clinical and endoscopic responses, with a good safety profile, within 8 weeks of a 2 gm/day QD treatment in adults with moderate UC. Two years later**,** the INDIGO study group (18) performed a multicenter randomized placebo-controlled trial in adults with UC. They showed a clinical remission rate of 55% with a 1 gm/day dose, and 38% with a 2 gm/day dose after 8 weeks of treatment. These proportions were significantly higher than those of the placebo. The high remission rate was demonstrated even in patients with steroid dependence, or with previous biological or immunomodulatory treatment, similar to some of our patients.

The first large QD study in children with UC (21) was conducted in Japan and included more than 100 patients. Clinical remission was shown for more than 80% within 6 months of treatment; the relapse rate was 64% in patients who used QD for more than 6 months. Seven patients experienced adverse events, which is a much higher rate than observed in the current study. The events included pulmonary hypertension in one patient, enteritis in five patients and headache in one.

Our management protocol included both curcumin and QD for 16 weeks, and then tapering of QD until discontinuation, while curcumin was continued. The advantage of our protocol is the prevention of QD side effects (13). In addition, the combined treatment enabled administrating lower doses of the two components, which act by different mechanisms and together provide a broader and stronger effect (15).

The study group reflects the pediatric UC population, characterized by active disease despite standard management. The multiple center design and prolonged follow-up period are strengths of the study. Nonetheless, the study has limitations stemming from the small sample size and the retrospective design, which limits the control over data completeness. Data were missing for some of the outcomes and the use of concurrent medications. In addition, endoscopic follow-up was performed predominantly in non-responding patients. This may have influenced the observed rate of mucosal healing, which contrasted with the findings of the adult study (15). We included patients with moderate disease who did not achieve complete clinical remission or who flared on corticosteroids or biological therapy. Those patients had mild-moderate active disease at the initiation of CurQD; however, their initial disease state was moderate as is evident also from the proportion that failed biologics. The remaining patients had a mild-moderate disease, which indicates heterogeneity of the patient population.

Our report of minimal adverse events suggests that CurQD has a good safety profile. However, due to the retrospective design, some side effects may not have been reported. In addition, as echocardiograms were not performed routinely, pulmonary hypertension, a known adverse event, may have occurred but not been reported.

In conclusion, our study shows that CurQD might be an effective and safe management for pediatric patients with mild to moderate UC who failed conventional management. Larger prospective studies are needed to evaluate the clinical and endoscopic effects of CurQD in children.

## Data Availability

The data analyzed in this study is subject to the following licenses/restrictions: retrospective data. Requests to access these datasets should be directed to batya.vais@sheba.health.gov.il.
